# Gait mechanics differences between healthy controls and patients with peripheral artery disease after adjusting for gait velocity stride length and step width.

**DOI:** 10.1123/jab.2017-0257

**Published:** 2018-07-10

**Authors:** John D. McCamley, Eric L. Cutler, Kendra K. Schmid, Shane R. Wurdeman, Jason M. Johanning, Iraklis I. Pipinos, Sara A. Myers

**Affiliations:** 1 Center for Research in Human Movement Variability, University of Nebraska at Omaha, Omaha, NE, USA;; 2 College of Public Health, University of Nebraska Medical Center, Omaha, NE, USA;; 3 Hanger Inc., Houston, TX, USA;; 4 Department of Surgery, Omaha VA Medical Center, Omaha, NE, USA;; 5 Department of Surgery, University of Nebraska Medical Center, Omaha, NE, USA

**Keywords:** intermittent claudication, gait biomechanics, joint torques and powers, walking velocity

## Abstract

Patients with peripheral artery disease (PAD) experience significant leg dysfunction. The effects of PAD on gait include shortened steps, slower walking velocity, and altered gait kinematics and kinetics, which may confound joint torques and power measurements.

Spatiotemporal parameters, joint torques and powers were calculated and compared between 20 patients with PAD and 20 healthy controls using independent t-tests. Separate ANCOVA models were used to evaluate group differences after independently adjusting for gait velocity, stride length and step width. Compared to healthy controls, patients with PAD exhibited reduced peak extensor and flexor torques at the knee, and hip. After adjusting for all covariates combined, differences between groups remained for ankle power generation in late stance, and knee flexor torque. Reduced walking velocity observed in subjects affected by PAD was closely connected with reductions in joint torques and powers during gait. Gait differences remained, at the knee and ankle, after adjusting for the combined effect of spatiotemporal parameters. Improving muscle function through exercise or with the use of assistive devices needs to be a key tool in the development of interventions that aim to enhance the ability of PAD patients to restore spatiotemporal gait parameters.

## Introduction

Peripheral artery disease (PAD) is a progressive disease caused by atherosclerotic blockages of the arteries supplying the legs.^1^ These blockages restrict blood flow to the affected legs and commonly present as claudication, exercise-induced cramping pain only relieved by rest. This presentation is known as intermittent claudication, and it can affect one or both legs.^2,3^ Patients with PAD, have reduced physical activity, and typically experience diminished mobility and quality of life.^1,2,4,5^ The American Heart Association, reports PAD currently affects in excess of 8 million Americans, including over 20% of those aged 65 years and over.^6^ The incidence of PAD is currently on the rise^7^ due to the prevalence of the underlying risk factors of PAD (e.g. high blood pressure, physical inactivity, obesity, high cholesterol and diabetes). As a growing public health concern, a more comprehensive understanding of PAD causal mechanisms is needed to promote improved functional outcomes.

A notable consequence of PAD is altered spatiotemporal gait parameters. Patients with PAD have been reported to walk with decreased step length, cadence and velocity and with increased stance time and step width compared to their healthy counterparts;^8^ and these are present from the first steps the patient takes before he or she experiences any claudication symptoms.^9,10^ Similarly, several studies have revealed altered or abnormal joint torques and powers, particularly at the ankle and hip, for PAD subjects, both with and without symptomatic claudication pain.^11–14^ More specifically, joint powers are reduced at the hip, knee and ankle in patients with PAD compared to velocity matched controls.^14^ The reduced peak powers and torques at the ankle in late stance that have been consistently reported in this pathological population^11–13^ inhibit patients with PAD from normal forward movement.^15^

Decreased ankle propulsion was suggested in one of the earliest studies to explore changes in gait mechanics in patients with PAD.^9^ This study found the presence of PAD resulted in slower walking velocities and shorter steps than age matched controls. A 2007 investigation^16^ reported flattened vertical ground reaction force curves, reduced forward propulsive forces and increased stance time in patients with PAD versus age matched controls during overground walking. In a subsequent study, patients with PAD exhibited increased peak ankle plantar flexion angle and range of motion during early stance.^17^ All reported effects were observed prior to claudication onset.

With multiple spatial-temporal, kinematic, and kinetic gait mechanics variables altered for patients with PAD it has proven difficult to distinguish their relationships with one another. Multiple studies have investigated the relationship between spatiotemporal gait variables and moments and powers during walking for healthy subjects.^18–20^ A study involving healthy older adults^21^ showed that spatiotemporal variables, such as stride length and gait velocity, were significant confounders for gait measurements. Studies have also observed the relationship between spatiotemporal changes and diseases such as osteoarthritis on kinematic and kinetic parameters.^22–25^ It is similarly possible that changes in spatiotemporal measures influence joint torque and power alterations in PAD gait.

Wurdeman et al.^14^ partially explored the effects of spatiotemporal measures by comparing limb joint torques and powers in patients with PAD to velocity matched controls. This study found no significant differences in joint torques at the ankle, knee, or hip. This contradicts previous work by the same group that did not account for differences in subjects’ preferred walking velocities, and reported differences in torques at the ankle, knee, and hip.^12,13^ Joint powers however, continued to show significant differences^14^ between the velocity matched groups. Ankle peak power generation during late stance, knee, peak power absorption in early and late stance, and hip peak power absorption in midstance, were reduced for patients with PAD. As was noted in the Wurdeman study,^14^ the procedure of matching patients with PAD to healthy controls based on walking velocity may have unintentionally suffered from selection bias, as those PAD subjects with faster self-selected walking velocities could be inherently patients with less severe disease manifestations.

The Wurdeman study,^14^ did not account for potential differences due to stride length or step width. Thus, the current study utilized a statistical approach to study the impact of spatiotemporal gait differences on kinematic and kinetic parameters. We determined whether differences in joint torques and powers at the ankle, knee, and hip, observed between PAD subjects and controls, was a true phenomenon or a secondary effect of changes in gait velocity, step length, and step width. We hypothesized that the spatiotemporal covariates were confounding variables of joint torques and powers between PAD subjects and controls. This study provided a more comprehensive understanding of the effects that confounding spatiotemporal measures have on gait in patients with PAD. Changes in statistical significance for a variable after adjusting for a co-varying spatiotemporal measure provided insight into the ultimate effect of altered joint torque or power.

## Methods

### Subjects:

All recruitment, screening, and testing was performed subsequent to approval by the relevant Institutional Review Boards. All subjects provided informed, written consent. Twenty healthy controls (age: 64.6 ± 8.0 years, height: 1.75 ± 0.08 m, mass: 83.8 ± 12.6 kg) and twenty bilateral PAD subjects (age: 62.6 ± 6.0 years, height: 1.78 ± 0.08 m, mass: 89.2 ± 15.5 kg) were enrolled in this study. Patients with PAD were diagnosed and screened for possible co-morbidities (neurological, musculoskeletal, and cardiopulmonary disorders) at a local vascular surgery clinic. Control subjects were recruited from the local community. Controls with age, height, and weight similar to the PAD group mean values were preferred. A personal medical history was collected, a physical examination and non-invasive ankle-brachial index screening was performed to confirm the presence or absence of PAD as well any possible co-morbidities. Subjects from the PAD and control groups were excluded if they required the use of an assistive device, such as a cane or walker.

### Data Collection:

Upon arrival to the laboratory for data collection, subjects were provided with a form fitting singlet and athletic footwear. This garment was necessary for the accurate marking of anatomical landmarks on the pelvis.^14^ Standardized athletic footwear was provided to maintain uniform sole shape and material properties. The subjects wore a Nike Dart men’s walking shoe of the appropriate size. Athletic footwear was used rather than barefoot because of the high risk of ulcer development in feet of patients with peripheral artery disease and to capture the mechanics of subjects with the constraints imposed in their typical environment. Height, weight, and age of the subjects, were recorded. Reflective markers were placed on the subject in a modified Helen Hayes marker set arrangement.^26^

Lower extremity kinetics were collected, at a rate of 600Hz, using a Kistler piezoelectric force platform (Kistler North America, Amherst, NY, USA) mounted flush with the surface of the floor. Lower extremity kinematics were collected at a rate of 60Hz using a Motion Analysis 8- camera motion capture system (Motion Analysis Corp., Santa Rosa, CA, USA).

All subjects walked along a clearly marked 10 m walkway at a self-selected pace. Five successful over-ground walking trials were collected for each leg. A successful trial was completed when the designated foot (left or right), landed completely within the area of the force plate. Subjects were required to rest for a minimum of one minute between trials in order to prevent overexertion and oxygen deprivation in the lower extremities. This ensured that PAD subjects did not experience claudication pain while walking.

### Data Analysis:

During subsequent processing, the marker position data was smoothed at a cutoff frequency of 6 Hz using a Butterworth fourth-order low pass filter. The positions of the reflective markers and ground reaction forces were utilized to calculate joint kinematics and kinetics during stance time for each trial. For patients with PAD the limb with the lower ankle-brachial index was utilized for kinetic analysis. For control subjects the right limb was chosen for analysis. The marker position data and ground reaction forces were analyzed using inverse dynamics as described by Vaughan et al^27^ and Nigg et al^28^ using Cortex (Motion Analysis Corp., Santa Rosa, CA) and Visual3D (C-Motion Inc., Germantown, MD) software. A custom MATLAB® (Mathworks Inc, Natick, MA) program was used to determine peak joint kinetics for each trial. Variables that were included for the analysis were the following peak joint torques: ankle dorsiflexor (ADT), ankle plantarflexor (APT), knee extensor (KET), knee flexor (KFT), hip extensor (HET), hip flexor (HFT). Dorsiflexion was defined as a movement of the forefoot toward the shank and plantarflexion was defined as a movement of the forefoot away from the shank. Flexion was defined as a decrease in the corresponding joint angle and extension was defined as an increase in the corresponding joint angle. Additionally, the following joint powers were calculated: ankle power absorption in early stance (A1), ankle power generation in late stance (A2), knee power absorption in early stance (K1), knee power generation in early stance (K2), knee power absorption in late stance (K3), hip power generation in early stance (H1), hip power absorption in mid-stance (H2) and hip power generation in late stance (H3).

### Statistical Analysis:

An independent t-test was used to compare joint torques and powers between controls and PAD subjects through univariate analysis. Separate ANCOVA models were used to evaluate the association of a condition with the outcome (joint torques or powers) adjusting for velocity, step width, and step length. An alpha value less than 0.05 was considered statistically significant.

## Results

The initial t-test found that control subjects walked significantly faster than patients with PAD (Table 1). The t-test also revealed significant differences for eight of the fourteen dependent variables. Significant differences in both peak extensor and flexor torque values were found at the knee and hip. Peak power differences were observed for ankle power generation in late stance, knee power absorption and generation during early stance, and knee power absorption during late stance.

Each of the three spatiotemporal variables was input individually as a covariate, as well as all three variables in combination. The inclusion of each of these covariates had an effect, on which of the dependent variables exhibited statistically significant differences (Table 2).

Adjusting for step width as a covariate had little effect on the variables that were significantly different. All previous differences observed for the univariate analysis (KET, KFT, HET, HFT, A2, K1, K2, and K3) remained significant, with an additional difference in peak ankle plantarflexor torque observed. Adjusting for stride length had a greater effect with significant differences no longer apparent for peak knee extensor torque or knee power absorption during early stance. The covariate which had the greatest effect on lower limb kinetic differences during stance was velocity. After adjusting for velocity, peak knee flexor torque and peak hip extensor torque and peak hip flexor torque, were significantly different between patients with PAD and the control subjects.

After adjusting for the combination all three spatiotemporal variables (stride length, step width and velocity) as covariates, the only significant differences that remained were for knee flexor torque and ankle power generation in late stance.

## Discussion

This study sought to examine whether known spatiotemporal differences in patients with PAD are driving gait alterations in peak joint torques and powers at the ankle, knee, and hip. Previously little work has been performed to determine how the changes in spatiotemporal variables that are observed for patients with PAD confound the changes in kinetic variables that are also observed during walking. We statistically controlled for gait velocity, step length, and step width differences between the patient with PAD and control groups. In our study, these covariates affected the statistical significance of certain gait variables, while most differences remained even after the adjustment.

Ankle power generation late in stance, in particular, was no longer different for PAD subjects compared to controls, after accounting for velocity alone. This would appear to confirm the role of ankle push off power in generating forward motion and suggests that a reduction in ankle power is at least a partial contributor to the reduction of walking velocity observed in patients with PAD. This was expected because power, by definition, is the product of angular velocity (rate of movement) and joint torque (force causing movement) during walking. As walking velocity decreases, the angular velocity of the ankle will likely decrease. It is important to note that angular velocities of the lower extremity joints are not the same as walking velocity. While some differences between groups in each of those rates is expected, it may not be proportionate to differences in walking velocity. Even though ankle power generation did not remain significantly different between PAD and controls after adjusting for walking velocity alone, it was different when adjusting for stride length alone, step width alone, and the combination of stride length, step width, and velocity. Considering there were no significant differences in stride lengths or step widths, but velocity was decreased in patients with PAD, our results suggest that patients with PAD have reduced ankle power generation compared with healthy individuals.

Accounting for velocity and stride length had inconsistent effects on torques at the knee. While peak knee extensor torques were no longer significantly different when the results were adjusted for velocity or stride length, significant differences in peak knee flexor torques remained after adjustment for all covariates. It appears that the observed differences in knee extensor torques during stance are at least in part a consequence of the lower walking velocity and reduced stride length of patients with PAD. This is not the case for knee flexor torques which remain significantly greater after adjustment for the combination of covariates. This is in accordance with the results reported by Lelas et al.^20^ who reported a strong relationship between knee flexor torque during stance and walking velocity, but not between knee extensor torque and velocity, for healthy subjects. These altered torques at the knee may also help explain the altered vertical ground reaction force profile during midstance observed previously,^16^ where patients with PAD had an increased vertical force during midstance.

Adjustment for walking velocity alone, removed the significant differences in all knee joint power peaks. This suggests that the lower powers measured at the knee account for lesser velocities observed in the PAD subjects.^20^ While reduced power absorption at the knee was observed in a study that utilized velocity matched subjects,^14^ reduced knee power absorption was not apparent when velocity was accounted for in this study. If the subjects with PAD in this study had reduced power absorption at the knee, this was masked by differing knee power requirements of altered walking velocity. It is possible that our previous study using velocity matched subjects led to selecting healthy controls who naturally walked slower and patients with PAD with naturally walked faster.^14^ It is also possible that twenty subjects per group is not adequate for investigating multiple confounders.

In this study hip torque differences between groups were not affected by controlling for step width or step length alone, but there were no longer differences in hip after controlling for the three combined covariates. We also did not observe differences in hip power requirements. This partially agrees with previous findings,^14^ which found no differences in hip power generation for velocity matched subjects.

It appears from these results that the ability of the distal lower limb musculature to generate propulsive power may be weakened by the presence of PAD. Such morphological and functional changes are supported through extensive research that has demonstrated muscle atrophy, mitochondrial dysfunction,^29–31^ and reduced limb strength.^32^ When patients with PAD pushed against a footplate,^33^ they were observed to have poorer leg extensor muscle power than subjects without PAD. An association has also been shown between lower ABI values and lower isometric plantarflexion strength, and knee extensor strength.^34^ It is possible this decreased muscle performance results in decreased walking velocity. It may also be that patients with PAD reduce their walking velocity to prolong the time to claudication, thus resulting in lower knee joint powers. It is difficult to directly detect which is the case, and it is also possible that both scenarios take place concurrently. Recent studies have demonstrated that when compared to healthy controls, joint powers are altered in individuals with PAD even before claudication is experienced.^8, 10^ This suggests that the affected limb might be physically limited from generating the powers required to walk at velocities similar to healthy controls, and that reduced walking velocity is not simply a strategy to avoid claudication.

This study controlled for the effect of velocity as well as stride length and step width mathematically, using the ANCOVA statistic. The statistical method of correcting for gait velocity had complementary effects as utilizing subjects with the same gait velocity. When comparing subsets of healthy subjects and patients with PAD that naturally walk at the same speed, there were no differences in joint torque variables, but multiple differences in powers. These results were most consistent with the current comparison that adjusted for all variables (velocity, stride length, and step width). In the other comparisons from this study, differences were found primarily for torque variables. Thus, the statistical approach can be complimentary and provide different insights regarding group differences and the impact of spatial temporal characteristics. Overall, this study reinforces previous studies emphasizing the importance of ankle function in late stance and knee torque as essential contributors to walking velocity. A more detailed investigation to determine how the timing of the altered joint torques and powers are interrelated, is necessary to fully understand the consequences muscle function changes due to PAD have on walking.

The results of this study show that the changes to spatiotemporal parameters of gait (stride length, step width, and velocity) observed in subjects affected by PAD are tightly interrelated with measurable joint torques and powers of the lower limbs during gait. This highlights the need to carefully interpret how measured changes in the spatial and temporal features of walking are explained by differing joint kinetics which are in turn caused by altered lower limb muscle function as a consequence of PAD. Many of the changes observed in gait characteristics of patients with PAD are already observed in older persons however to a greater degree. It has already been shown that aging causes a proximal change in joint torques and powers.^35^ PAD has an effect on the distal musculature of the lower limbs so it is no surprise that it will cause gait changes similar to, but of greater magnitude than, those already observed in the elderly. This study highlights the importance of taking into consideration secondary changes to gait patterns when assessing the effect of pathologies that effect walking mechanics. Future studies are necessary to further determine how PAD changes specific muscle function in the lower limbs and limits the ambulation of PAD patients. Improving muscle function through exercise or with the use of assistive devices needs to be a key tool in the development of any intervention that aims to enhance the ability of PAD patients to restore spatiotemporal gait parameters.

## Figures and Tables

**Table 1:** Univariate comparison between patients with PAD and healthy age-matched controls.

Dependent Variable	Control Mean (SD)	PAD Mean (SD)	T-test *p*-value
Stride length (mm)	1488 (113)	1428 (87)	.069
Step width (mm)	112 (27)	120 (22)	.278
Velocity (m.s^−1^)	1.32 (0.12)	1.24 (0.07)	**.023** *
ADT	0.35 (0.09)	0.30 (0.11)	.152
APT	1.46 (0.21)	1.35 (0.12)	.056
KET	0.78 (0.25)	0.59 (0.20)	**.013** *
KFT	0.11 (0.13)	0.20 (0.10)	**.018** *
HET	0.92 (0.22)	0.74 (0.24)	**.019** *
HFT	1.10 (0.22)	0.94 (0.18)	**.018** *
A1	0.83 (0.26)	0.76 (0.30)	.420
A2	2.89 (0.82)	2.27 (0.33)	**.005** *
K1	1.11 (0.55)	0.78 (0.30)	**.028** *
K2	0.57 (0.32)	0.35 (0.19)	**.011** *
K3	1.30 (0.61)	0.91 (0.28)	**.016** *
H1	0.69 (0.30)	0.34 (0.20)	.104
H2	0.90 (0.24)	0.75 (0.24)	.054
H3	0.99 (0.20)	0.87 (0.22)	.077

All variables are peak values. ADT: ankle dorsiflexor torque, APT: ankle plantarflexor torque, KET: knee extensor torque, KFT: knee flexor torque, HET: hip extensor torque, HFT: hip flexor torque, A1: ankle power absorption in mid-stance, A2: ankle power generation in late stance, K1: knee power absorption in early stance, K2: knee power generation in early stance, K3: knee power absorption in late stance , H1: hip power generation in early stance , H2: hip power absorption in mid-stance, H3: hip power generation in late stance.

Note: All torque are reported in N.m.kg^−1^ power in W.kg^−1^. Stride length and step width in mm and velocity in m/s.

* and bold type indicates a significant difference (*p* < 0.05) between groups

**Table 2:** Significance (p-value) level for four independent ANCOVA analyses. The comparisons between healthy controls and patients with PAD were statistically adjusted for stride length, step width, and walking velocity independently and all three combined (All).

Covariate	All	Stride length	Step width	Velocity
ADT	.944	.468	.271	.573
APT	.137	.281	**.024** *	.257
KET	.177	.059	**.019** *	.120
KFT	**.041** *	**.013** *	**.032** *	**.025** *
HET	.066	**.031** *	**.027** *	**.045** *
HFT	.074	**.036** *	**.025** *	**.050** *
A1	.139	.414	.331	.174
A2	**.044** *	**.017** *	**.003** *	.062
K1	.273	.130	**.029** *	.241
K2	.118	**.048** *	**.012** *	.101
K3	.132	**.026** *	**.023** *	.155
H1	.111	.087	.090	.136
H2	.175	.130	.063	.143
H3	.254	.091	.088	.285

Peak values of dependent variables are reported. ADT: ankle dorsiflexor torque, APT: ankle plantarflexor torque, KET: knee extensor torque, KFT: knee flexor torque, HET: hip extensor torque, HFT: hip flexor torque, A1: ankle power absorption in mid-stance, A2: ankle power generation in late stance, K1: knee power absorption in early stance, K2: knee power generation in early stance, K3: knee power absorption in late stance , H1: hip power generation in early stance , H2: hip power absorption in mid-stance, H3: hip power generation in late stance.

* and bold type indicates a significant difference (*p* < 0.05) between groups

## References

[1] Hallett JW. Peripheral Arterial Disease. Am J Respir Crit Care Med. 2008;168(4):425–430. doi:10.1161/01.CIR.0000160581.58633.8B.

[2] McDermott MM, Greenland P, Liu K, Leg Symptoms in Peripheral Arterial Disease. JAMA. 2001;286(13):1599. doi:10.1001/jama.286.13.1599. 11585483

[3] Norgren L, Hiatt WR. Inter-Society Consensus for the Management of Peripheral Arterial Disease. Eur J Vasc Endovasc Surg. 2007;70(Tasc Ii):1–73. doi:10.1016/j.ejvs.2006.09.024. 17140820

[4] Gardner AW, Parker DE, Montgomery PS, Blevins SM, Teague AM, Casanegra AI. Monitored Daily Ambulatory Activity, Inflammation, and Oxidative Stress in Patients With Claudication. Angiology. 2013;65(6):491–496. doi:10.1177/0003319713489769. 23695338 PMC3841224

[5] Gardner AW, Montgomery PS, Scott KJ, Afaq A, Blevins SM. Patterns of ambulatory activity in subjects with and without intermittent claudication. J Vasc Surg. 2007;46(6):1208–1214. doi:10.1016/j.jvs.2007.07.038. 17919876 PMC2222553

[6] Roger VL, Go AS, Lloyd-Jones DM, Heart Disease and Stroke Statistics−−2011 Update: A Report From the American Heart Association. Circulation. 2011;123(4):e18–e209. doi:10.1161/CIR.0b013e3182009701. 21160056 PMC4418670

[7] Creager MA, Belkin M, Bluth EI, 2012 ACCF/AHA/ACR/SCAI/SIR/STS/SVM/SVN/SVS Key Data Elements and Definitions for Peripheral Atherosclerotic Vascular Disease. J Am Coll Cardiol. 2012;59(3):294–357. doi:10.1016/j.jacc.2011.10.860. 22153885

[8] Wurdeman SR, Myers SA, Johanning JM, Pipinos II, Stergiou N. External work is deficient in both limbs of patients with unilateral PAD. Med Eng Phys. 2012;34(10):1421–1426. doi:10.1016/j.medengphy.2012.01.004. 22321812 PMC3360992

[9] Scherer SA, Bainbridge JS, Hiatt WR, Regensteiner JG. Gait characteristics of patients with claudication. Arch Phys Med Rehabil. 1998;79(5):529–531. doi:10.1016/S0003-9993(98)90067-3. 9596393

[10] McCully K, Leiper C, Sanders T, Griffin E. The effects of peripheral vascular disease on gait. Journals Gerontol Ser A Biol Sci Med Sci. 1999;54(7):B291–B294. doi:10.1093/gerona/54.7.B291. 10462161

[11] Chen SJ, Pipinos I, Johanning J, Bilateral claudication results in alterations in the gait biomechanics at the hip and ankle joints. J Biomech. 2008;41(11):2506–2514. doi:10.1016/j.jbiomech.2008.05.011. 18586253

[12] Koutakis P, Johanning JM, Haynatzki GR, Abnormal joint powers before and after the onset of claudication symptoms. J Vasc Surg. 2010;52(2):340–347. doi:10.1016/j.jvs.2010.03.005. 20670775 PMC2921796

[13] Koutakis P, Pipinos II, Myers SA, Stergiou N, Lynch TG, Johanning JM. Joint torques and powers are reduced during ambulation for both limbs in patients with unilateral claudication. J Vasc Surg. 2010;51(1):80–88. doi:10.1016/j.jvs.2009.07.117. 19837536 PMC4465243

[14] Wurdeman SR, Koutakis P, Myers SA, Johanning JM, Pipinos II, Stergiou N. Patients with peripheral arterial disease exhibit reduced joint powers compared to velocity-matched controls. Gait Posture. 2012;36(3):506–509. doi:10.1016/j.gaitpost.2012.05.004. 22677467 PMC3407282

[15] Meinders M, Gitter A, Czerniecki JM. The role of ankle plantar flexor muscle work during walking. Scand J Rehabil Med. 1998;30(1):39–46. doi:10.1080/003655098444309. 9526753

[16] Scott-Pandorf MM, Stergiou N, Johanning JM, Robinson L, Lynch TG, Pipinos II. Peripheral arterial disease affects ground reaction forces during walking. J Vasc Surg. 2007;46(3):491–499. doi:10.1016/j.jvs.2007.05.029. 17826236

[17] Celis R, Pipinos II, Scott-Pandorf MM, Myers SA, Stergiou N, Johanning JM. Peripheral arterial disease affects kinematics during walking. J Vasc Surg. 2009;49(1):127–132. doi:10.1016/j.jvs.2008.08.013. 19028062

[18] Winter DA. Biomechanical motor patterns in normal walking. J Mot Behav. 1983;15(4):302–330. doi:10.1080/00222895.1983.10735302. 15151864

[19] Kirtley C, Whittle MW, Jefferson RJ. Influence of walking speed on gait parameters. J Biomed Eng. 1985;7(4):282–288. doi:10.1016/0141-5425(85)90055-X. 4057987

[20] Lelas JL, Merriman GJ, Riley PO, Kerrigan DC. Predicting peak kinematic and kinetic parameters from gait speed. Gait Posture. 2003;17(2):106–112. doi:10.1016/S0966-6362(02)00060-7. 12633769

[21] Elble RJ, Thomas SS, Higgins C, Colliver J. Stride-dependent changes in gait of older people. J Neurol. 1991;238(1):1–5. doi:10.1007/BF00319700. 2030366

[22] Olney SJ, Griffin MP, McBride ID. Temporal, kinematic, and kinetic variables related to gait speed in subjects with hemiplegia: a regression approach. Phys Ther. 1994;74(9):872–885. http://www.atsjournals.org/doi/abs/10.1164/rccm.200208-856OC. 8066114 10.1093/ptj/74.9.872

[23] Landry SC, McKean KA, Hubley-Kozey CL, Stanish WD, Deluzio KJ. Knee biomechanics of moderate OA patients measured during gait at a self-selected and fast walking speed. J Biomech. 2007;40(8):1754–1761. doi:10.1016/j.jbiomech.2006.08.010. 17084845

[24] Astephen Wilson JL. Challenges in dealing with walking speed in knee osteoarthritis gait analyses. Clin Biomech. 2012;27(3):210–212. doi:10.1016/j.clinbiomech.2011.09.009. 22019141

[25] Zeni JA, Higginson JS. Differences in gait parameters between healthy subjects and persons with moderate and severe knee osteoarthritis: A result of altered walking speed? Clin Biomech. 2009;24(4):372–378. doi:10.1016/j.clinbiomech.2009.02.001. PMC2715920 19285768

[26] Houck J, Yack HJ, Cuddeford T. Validity and comparisons of tibiofemoral orientations and displacement using a femoral tracking device during early to mid stance of walking. Gait Posture. 2004;19(1):76–84. doi:10.1016/S0966-6362(03)00033-X. 14741306

[27] Vaughan CL, Davis BL, O’Connor JC. The Three-Dimensional and Cyclic Nature of Gait. Vaughan CL, ed. Dyn Hum Gait. 1999;168(4):16–17. http://www.atsjournals.org/doi/abs/10.1164/rccm.200208-856OC.

[28] Nigg BM, Cole GK, Nachbauer W. Effects of arch height of the foot on angular motion of the lower extremities in running. J Biomech. 1993;26(8):909–916. doi:10.1016/0021-9290(93)90053-H. 8349716

[29] Pipinos II, Judge AR, Selsby JT, The Myopathy of Peripheral Arterial Occlusive Disease: Part 1. Functional and Histomorphological Changes and Evidence for Mitochondrial Dysfunction. Vasc Endovascular Surg. 2008;41(6):481–489. doi:10.1177/1538574407311106. 18166628

[30] Pipinos II, Judge AR, Selsby JT, The myopathy of peripheral arterial occlusive disease: Part 2. Oxidative stress, neuropathy, and shift in muscle fiber type. Vasc Endovascular Surg. 2008;42(2):101–112. doi:10.1177/1538574408315995. 18390972 PMC12282609

[31] McDermott MM. Lower Extremity Manifestations of Peripheral Artery Disease: The Pathophysiologic and Functional Implications of Leg Ischemia. Circ Res. 2015;116(9):1540–1550. doi:10.1161/CIRCRESAHA.114.303517. 25908727 PMC4410164

[32] McDermott MM, Criqui MH, Greenland P, Leg strength in peripheral arterial disease: Associations with disease severity and lower-extremity performance. J Vasc Surg. 2004;39(3):523–530. doi:10.1016/j.jvs.2003.08.038. 14981443

[33] McDermott MM, Guralnik JM, Albay M, Bandinelli S, Miniati B, Ferrucci L. Impairments of muscles and nerves associated with peripheral arterial disease and their relationship with lower extremity functioning: the InCHIANTI Study. J Am Geriatr Soc. 2004;52:405–410. 14962156 10.1111/j.1532-5415.2004.52113.x

[34] McDermott MM, Tian L, Ferrucci L, Associations Between Lower Extremity Ischemia, Upper and Lower Extremity Strength, and Functional Impairment with Peripheral Arterial Disease. J Am Geriatr Soc. 2008;56(4):724–729. doi:10.1111/j.1532-5415.2008.01633.x. 18284536 PMC2645633

[35] DeVita P, Hortobagyi T. Age causes a redistribution of joint torques and powers during gait. J Appl Physiol. 2000;88(5):1804–1811. http://www.atsjournals.org/doi/abs/10.1164/rccm.200208-856OC. 10797145 10.1152/jappl.2000.88.5.1804

